# Machine learning for risk stratification of in-hospital mortality in patients with cervical cancer: an analysis of hospital admissions in Mato Grosso, Brazil

**DOI:** 10.1186/s12885-026-16771-z

**Published:** 2026-08-18

**Authors:** Audêncio Victor, Sancho Xavier Pedro, Fabiano Barcellos Filho, Ageo Mario Cândido da Silva, Vanilda Alves Moreira, Cornélio Victor, Patrícia H.C. Rondó, Alexandre Dias Porto Chiavegatto Filho

**Affiliations:** 1 School of Public Health, University of São Paulo (USP), Av. Dr. Arnaldo, 715, Cerqueira César, São Paulo, 01246-904 Brazil; 2 Institute of Collective Health, Federal University of Mato Grosso, Av. Fernando Corrêa da Costa, 2367, Boa Esperança, Cuiabá, Mato Grosso 78060-900 Brazil; 3 Department of Statistics, Centre of Exact Sciences, State University of Maringá, Avenida Colombo, 5790, Bloco E90, Zona 07, Maringá, Paraná CEP 87020-900 Brasil; 4 Faculty of Epidemiology and Population Health, London School of Hygiene and Tropical Medicine, Keppel Street, London, WC1E 7HT UK; 5 School of Public Health, University of São Paulo (USP), Avenida Doutor Arnaldo, 715, São Paulo, São Paulo 01246904 Brazil

**Keywords:** Cervical cancer, Hospital mortality, Machine learning, Oncology, Mato Grosso

## Abstract

**Introduction:**

Cervical cancer (CC) remains a significant cause of cancer mortality among women in low- and middle-income countries, particularly in regions with limited healthcare resources. This study aims to develop and evaluate machine learning (ML) models for hospital-level risk stratification of in-hospital mortality among patients hospitalized with CC in Mato Grosso, Brazil, between 2011 and 2023.

**Methods:**

Hospitalization data for CC patients were obtained from the SUS Hospital Information System (SIH). The dataset included demographic, clinical, and hospital variables. Five ML algorithms: Logistic Regression, Random Forest, CatBoost, LightGBM, and XGBoost, were implemented. Model performance was evaluated using AUC-ROC, calibration (Brier score), and threshold-dependent measures (accuracy, sensitivity, specificity, precision, and F1-score). Model interpretability was examined using Shapley Additive Explanations (SHAP).

**Results:**

A total of 3,493 hospitalisations were analysed. Among the evaluated models, XGBoost achieved the best overall performance, with an AUC-ROC of 0.89, accuracy of 88%, and specificity of 95%, and demonstrated good calibration (Brier score 0.08). Tree-based models, including Random Forest and CatBoost, also showed high specificity (> 93%) and low Brier scores. SHAP analysis identified medical procedure type, hospitalization cost, and service complexity as the most influential predictors of in-hospital mortality.

**Conclusion:**

ML models demonstrated robust performance in estimating in-hospital mortality risk among cervical cancer patients using administrative hospitalization data. By combining predictive performance with model interpretability, these approaches provide insights into clinical and system-level factors associated with mortality and may support hospital-level risk stratification, health system evaluation, and resource allocation in resource-limited settings.

**Supplementary Information:**

The online version contains supplementary material available at https://doi.org/10.1186/s12885-026-16771-z.

## Introduction

Cervical cancer (CC) is a leading cause of cancer mortality among women in low- and middle-income countries, with approximately 300,000 deaths annually, 85% of which occur in developing regions [1, 2]. In Brazil, CC ranks third in incidence among female cancers, excluding non-melanoma skin cancers, with an estimated 17,010 new cases annually between 2023 and 2025, corresponding to an incidence rate of 15.38 per 100,000 women [3]. Despite the implementation of screening programs and HPV vaccination efforts, mortality remains high in regions with limited access to high-complexity healthcare services [4, 5].

The state of Mato Grosso, Brazil, exemplifies the challenges faced in combating CC, with vast rural and indigenous areas where specialised healthcare access is limited, restricting opportunities for early diagnosis and adequate treatment [10]. Regional disparities in treatment access, coupled with factors such as poverty and limited education, create a high-risk environment for women in this area [6–8]. Additional determinants, including advanced age at diagnosis, comorbidities, and limited availability of intensive care, are strongly associated with poor clinical outcomes [6, 9]. Prior studies have underscored the importance of factors such as age, cancer stage at diagnosis, and treatment type in determining CC prognosis [6, 9–11]. While survival analysis has provided valuable insights into risk factors and survival rates in this population, there is a pressing need for more advanced analytical methods capable of integrating complex datasets and identifying nonlinear patterns [10].

In recent years, advancements in precision medicine and artificial intelligence (AI) have driven the adoption of ML techniques, which excel in uncovering intricate relationships within large datasets. ML algorithms have shown significant promise in various medical domains, from predicting recurrence to stratifying risks in cancer patients [12–15]. However, the application of ML in predicting hospital mortality for CC patients remains underexplored, particularly in low-resource settings where logistical and infrastructural challenges hinder technological innovation [15–17]. ML offers significant advantages over traditional methods by processing large volumes of data and identifying subtle predictive factors [17]. Unlike conventional survival models, which often rely on linear assumptions, ML models effectively capture nonlinear relationships and complex interactions between variables.

Recent studies have demonstrated ML’s potential in cancer prognosis. For instance, Rahimi et al. (2023) highlighted ML’s superiority over traditional methods in predicting cervical cancer survival outcomes, emphasizing its ability to integrate diverse clinical and demographic variables for improved accuracy [18, 19]. Additionally, advancements in model interpretability, such as Shapley Additive Explanations (SHAP), have addressed a key barrier to clinical adoption. SHAP enhances transparency by quantifying the contribution of individual predictors, enabling actionable insights for healthcare professionals [20].

Despite its benefits, SHAP-based interpretability in CC-related research remains underutilised, particularly in predicting hospital mortality where clinical, demographic, and socioeconomic factors interplay. Bridging this gap is critical to supporting evidence-based decision-making, especially in resource-limited environments. Therefore, this study aims to develop and evaluate ML models for hospital-level risk stratification of in-hospital mortality among patients hospitalised with cervical cancer in Mato Grosso, Brazil, between 2011 and 2023, using hospitalization-episode-level administrative data.

## Methods

### Dataset description

This retrospective observational study utilised data from the SUS Hospital Information System (SIH), encompassing hospitalizations of CC-diagnosed patients in Mato Grosso, Brazil, from 2011 to 2023. Data were sourced from the Data Repository of the State Health Secretariat of Mato Grosso (SES-MT), part of SUS. SIH, established in 1991, is an administrative database recording hospital admission in SUS through the Hospitalization Authorization form (AIH), primarily for reimbursement of hospital expenses and providing data for morbidity and mortality studies [21]. These data are publicly accessible online at: http://appweb3.saude.mt.gov.br/dw/pesquisa/tema. Ethical approval was not required, as all procedures adhered to scientific research ethics and respected data privacy and confidentiality [22].

### Variables

Analysed characteristics included patient age groups (16-39, 40-59, and 60 + years), race, residence municipality, and cancer type by topography, categorized into malignant neoplasm of the cervix (C53), unspecified cervix (C53.9), ectocervix (C53.1), endocervix (C53.0), and invasive lesions (C53.8). “Unspecified” and “not otherwise classified” (NOS) categories were combined, as both indicate a lack of specific tumor location details. Medical procedures during hospitalisation were classified as clinical or surgical, and the hospitalisation period was categorized by whether it occurred during or outside the COVID-19 pandemic. Hospitalisation type was classified as urgent or elective, and service complexity was categorised as medium or high. Other factors included ICU admission and total hospitalisation costs, adjusted to USD, and compared to the median.

The outcome in this study was hospital mortality, defined as in-hospital death. Patients were categorised as “died” or “not died” based on hospital mortality records from the SIH database.

### Machine learning model design

To predict hospital mortality in CC patients, we applied a diverse set of ML algorithms to explore different predictive approaches and compare their performance. Model selection and training methodology were based on best practices for binary classification problems in clinical settings.

### Data preprocessing

Continuous variables were standardised using z-scores, and categorical variables were encoded using one-hot encoding. Predictors with more than 20% missing values were excluded. For variables with fewer than 20% missing values, mean imputation was performed using statistics derived exclusively from the training dataset, and the same parameters were subsequently applied to the test dataset to avoid data leakage, in accordance with prior recommendations for handling missing data in healthcare datasets [23, 24]. To ensure unbiased evaluation of model performance, the dataset was randomly split into a training set (80%) and an independent test set (20%). Within the training set, 10-fold cross-validation was applied for hyperparameter tuning and internal model selection, while the test set was held out exclusively for the final assessment of discrimination and calibration. This nested strategy was preferred over relying solely on 10-fold cross-validation because, given the moderate sample size and the 9.9% in-hospital mortality rate, preserving a partition that was never used during model development or tuning reduces the risk of optimistic performance estimates and is consistent with TRIPOD recommendations for internal validation of prediction models.

### Algorithm selection

Five machine learning algorithms were tested: CatBoost [25], XGBoost [26], LightGBM [27], Random Forest and Logistic Regression. These algorithms were selected for their demonstrated robustness in classification tasks within healthcare settings. CatBoost, XGBoost, and LightGBM: These gradient-boosting algorithms are well-suited for datasets with heterogeneous features and missing values. Random Forest: A robust ensemble learning method known for its interpretability and ability to handle high-dimensional data. Logistic Regression: Included as a baseline model for comparison, given its traditional use in healthcare prediction tasks. Python packages specific to each algorithm were utilised, while scikit-learn was employed for Random Forest and Logistic Regression [28].

### Hyperparameter tuning

Hyperparameters were selected via 10-fold cross-validation using Bayesian optimisation and RandomSearch [29]. Given the observed class imbalance in the dataset, with in-hospital mortality accounting for 9.9% of hospitalisations, the Synthetic Minority Over-sampling Technique (SMOTE) was applied to the training data during model development. Bayesian optimisation was chosen for its efficiency in finding optimal hyperparameter configurations in large search spaces, leveraging probabilistic modelling to focus on promising regions. Random Search was used to complement this approach, given its simplicity and effectiveness in exploring diverse configurations. Grid Search was avoided due to its computational intensity and limited scalability when dealing with high-dimensional hyperparameter spaces.

### Feature selection

Feature selection was conducted using the BORUTA method, which iteratively identifies the most relevant predictors by comparing their importance against randomly permuted shadow features. This method ensured that only the most predictive variables were retained in the training set [30].

### Model evaluation

Model performance was evaluated on the independent test set using discrimination metrics, including the area under the receiver operating characteristic curve (AUC-ROC), as well as threshold-dependent measures (accuracy, sensitivity, specificity, precision, and F1-score). Model calibration was assessed using the Brier score with 95% confidence intervals, providing a quantitative measure of agreement between predicted probabilities and observed outcomes. The model with the best performance and calibration was subsequently analysed using Shapley Additive Explanations (SHAP) [31–33], to quantify the contribution of individual predictors to the model outputs. SHAP values represent the average marginal contribution of each feature to a prediction, providing a theoretically consistent and model-agnostic explanation of how individual variables influence the predicted probability of in-hospital mortality; positive values indicate an increase and negative values a decrease in predicted risk, allowing assessment of both the direction and magnitude of each variable’s effect. The overall reporting and evaluation strategy adheres to the Transparent Reporting of a Multivariable Prediction Model for Individual Prognosis or Diagnosis (TRIPOD) guidelines and relevant extensions for machine learning–based prediction models [34]. In addition to the Brier score, model calibration was examined graphically using calibration curves (reliability diagrams), both for the original models and after post-hoc isotonic recalibration fitted on a held-out calibration subset of the training data. The potential clinical utility of the models was further assessed using decision curve analysis (DCA), which quantifies the net benefit of model-guided decisions across a range of threshold probabilities (Supplementary Figs. S5 and S6).

## Results

A total of 3,493 CC hospitalisations were included in the analysis, of which 3,148 (90.1%) resulted in survival and 345 (9.9%) resulted in in-hospital death. Survival rates were lowest among patients aged 60 years and older, with 598 (83.5%) surviving. In contrast, the highest survival rates were observed among patients undergoing surgical procedures 2,149 (98.4%) and those treated in high-complexity healthcare facilities 1,006 (99.3%).

Patients diagnosed with ectocervix cancer exhibited the most favourable outcomes, with 331 (98.8%) surviving. During the COVID-19 pandemic period, survival was slightly lower 463 (88.4%) compared with the non-pandemic period 2,685 (90.4%). ICU admission was strongly associated with poorer outcomes, with survival observed in 213 (72.0%), compared with 2,935 (91.8%) who did not require ICU care (Table 1).

**Table 1:** Demographic and clinical characteristics of cervical cancer patients hospitalized in Mato Grosso, Brazil (2011–2023)

Variable	Total (*n* = 3493)	Died	
		No (*n* = 3148)	Yes (*n* = 345)
Age (years)			
16–39	1147 (32.8%)	1083 (94.4%)	64 (5.6%)
40–59	1630 (46.7%)	1467 (90.0%)	163 (10.0%)
60 and over	716 (20.5%)	598 (83.5%)	118 (16.5%)
Race/Skin colour			
White	634 (18.2%)	579 (91.3%)	55 (8.7%)
Non-White	2859 (81.8%)	2569 (89.9%)	290 (10.1%)
City of residence			
Cuiabá	825 (23.6%)	734 (89.0%)	91 (11.0%)
Rondonópolis	312 (8.9%)	265 (84.9%)	47 (15.1%)
Sinop	221 (6.3%)	209 (94.6%)	12 (5.4%)
Várzea Grande	353 (10.1%)	303 (85.8%)	50 (14.2%)
Others	1782 (51.0%)	1637 (91.9%)	145 (7.9%)
Type of cancer by topography			
NOS/Unspecified	1452 (41.6%)	1250 (86.1%)	202 (13.9%)
Ectocervix	335 (9.6%)	331 (98.8%)	4 (1.2%)
Endocervix	885 (25.3%)	861 (97.3%)	24 (2.7%)
Invasive Lesion	821 (23.5%)	706 (86.0%)	115 (14.0%)
Medical procedure			
Surgical	2183 (62.5%)	2149 (98.4%)	34 (1.6%)
Non-surgical	1310 (37.5%)	999 (76.3%)	311 (23.7%)
Hospitalization period			
Non-pandemic	2969 (85.0%)	2685 (90.4%)	284 (9.6%)
Pandemic	524 (15.0%)	463 (88.4%)	61 (11.6%)
Admission type			
Urgent/Emergency	1700 (48.7%)	1624 (95.5%)	76 (4.5%)
Elective	1793 (51.3%)	1524 (85.0%)	269 (15.0%)
Service complexity			
High	1013 (29.0%)	1006 (99.3%)	7 (0.7%)
Medium	2480 (71.0%)	2142 (86.4%)	338 (13.6%)
ICU admission			
No	3197 (91.5%)	2935 (91.8%)	262 (8.2%)
Yes	296 (8.5%)	213 (72.0%)	83 (28.0%)
Cost of hospitalization (mean = 152,971 USD)			
Up to median	1722 (49.3%)	1517 (88.1%)	205 (11.9%)
Above median	1771 (50.7%)	1631 (92.1%)	140 (7.9%)

### Predictive model performance

**Fig. 1:**
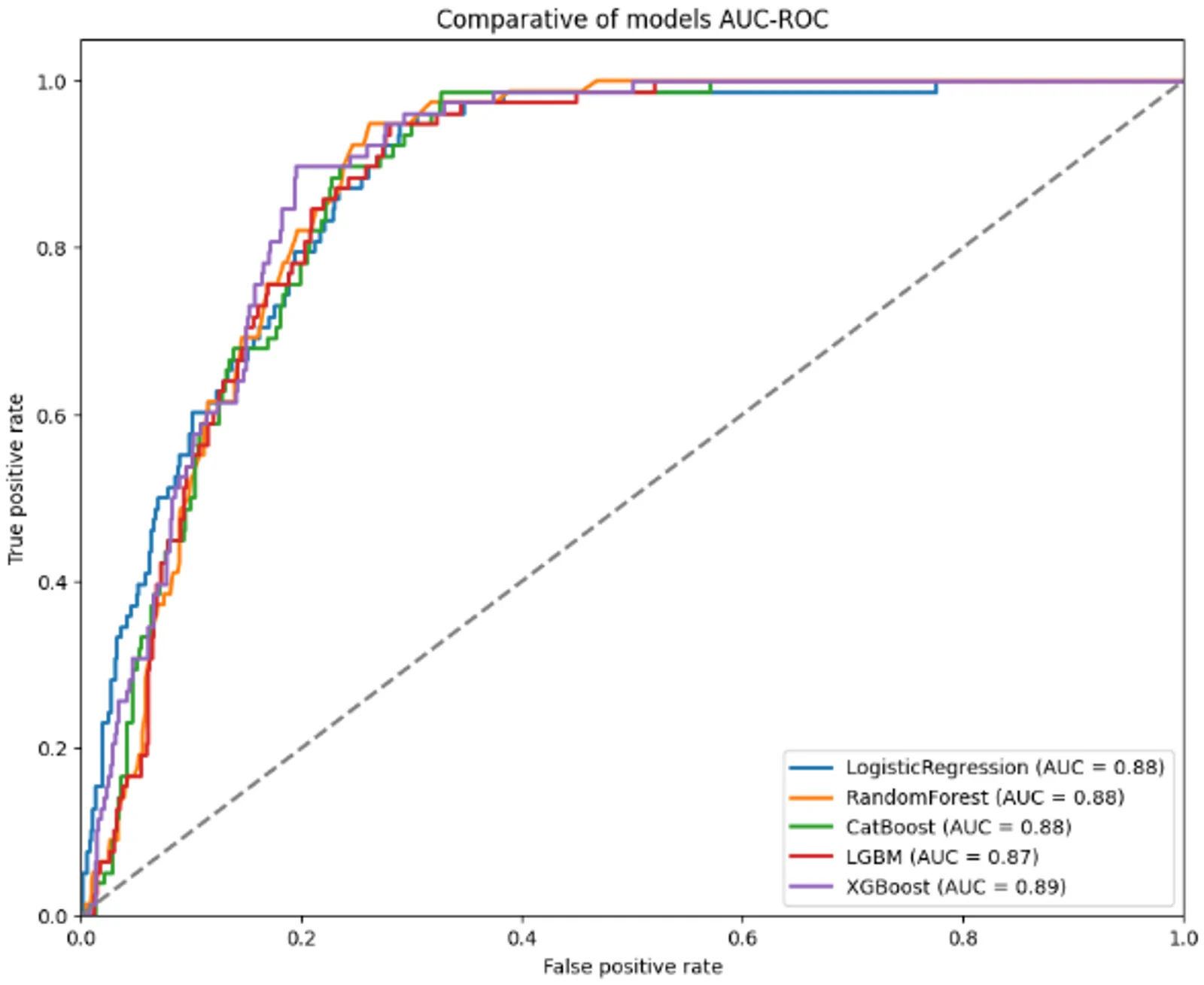
Predictive performance on test data of algorithms for each model in terms of AUC-ROC

The performance of the models is summarised in Table 2; Fig. 1. All machine learning models showed good discriminative performance, with AUC-ROC values ranging from 0.87 to 0.89. Among the tested algorithms, XGBoost achieved the highest discrimination, with an AUC-ROC of 0.89 (95% CI 0.86-0.91). Random Forest and CatBoost showed similar discrimination (AUC-ROC ≈ 0.88), while LightGBM achieved an AUC-ROC of 0.87 (95% CI 0.84-0.90). In terms of calibration, tree-based models showed lower Brier scores, indicating better probabilistic accuracy. XGBoost and Random Forest achieved the lowest Brier scores (0.08; 95% CI 0.07-0.10), followed by CatBoost (0.09; 95% CI 0.08-0.11) and LightGBM (0.09; 95% CI 0.08-0.11), whereas Logistic Regression showed a higher Brier score (0.14; 95% CI 0.12-0.15).

**Table 2:** Predictive performance on test data of the best algorithm for each outcome with hyperparameter tuning

Model	AUC-ROC (95% CI)	Brier score (95% CI)	Accuracy	Sensitivity	Specificity	Precision	F1-score
Logistic Regression	0.88 (0.85-0.91)	0.14 (0.12-0.15)	0.79	0.82	0.78	0.29	0.43
Random Forest	0.88 (0.85-0.90)	0.08 (0.07-0.10)	0.88	0.32	0.94	0.35	0.34
CatBoost	0.88 (0.85-0.90)	0.09 (0.08-0.11)	0.88	0.33	0.94	0.38	0.36
LightGBM	0.87 (0.84-0.90)	0.09 (0.08-0.11)	0.87	0.22	0.94	0.28	0.25
XGBoost	0.89 (0.86-0.91)	0.08 (0.07-0.10)	0.88	0.31	0.95	0.39	0.34

Discrimination, calibration, and threshold-based performance metrics of machine learning models for predicting in-hospital mortality among cervical cancer patients using administrative hospitalisation data. Threshold-dependent metrics were calculated using a fixed probability cutoff of 0.5

At a fixed probability threshold of 0.5, XGBoost achieved an accuracy of 88%, sensitivity of 31%, specificity of 95%, precision of 39%, and an F1-score of 0.34. CatBoost and Random Forest demonstrated comparable accuracy (88%) and high specificity (> 93%), with sensitivities of 33% and 32%, respectively. Logistic Regression showed higher sensitivity (82%) but lower specificity (78%) and precision (29%) (Table 2). The 0.5 cut-off is reported as the conventional default decision threshold, providing a familiar reference point for the confusion-matrix-based metrics. Because in-hospital mortality was infrequent (9.9%), this threshold favours specificity over sensitivity and is not necessarily the operating point that maximises detection of deaths; the full range of thresholds is therefore reported in Supplementary Table S1, and the consequences of threshold choice for clinical decision-making are explored through decision curve analysis (Supplementary Fig. S6).

### SHAP-Based predictor analysis

**Fig. 2:**
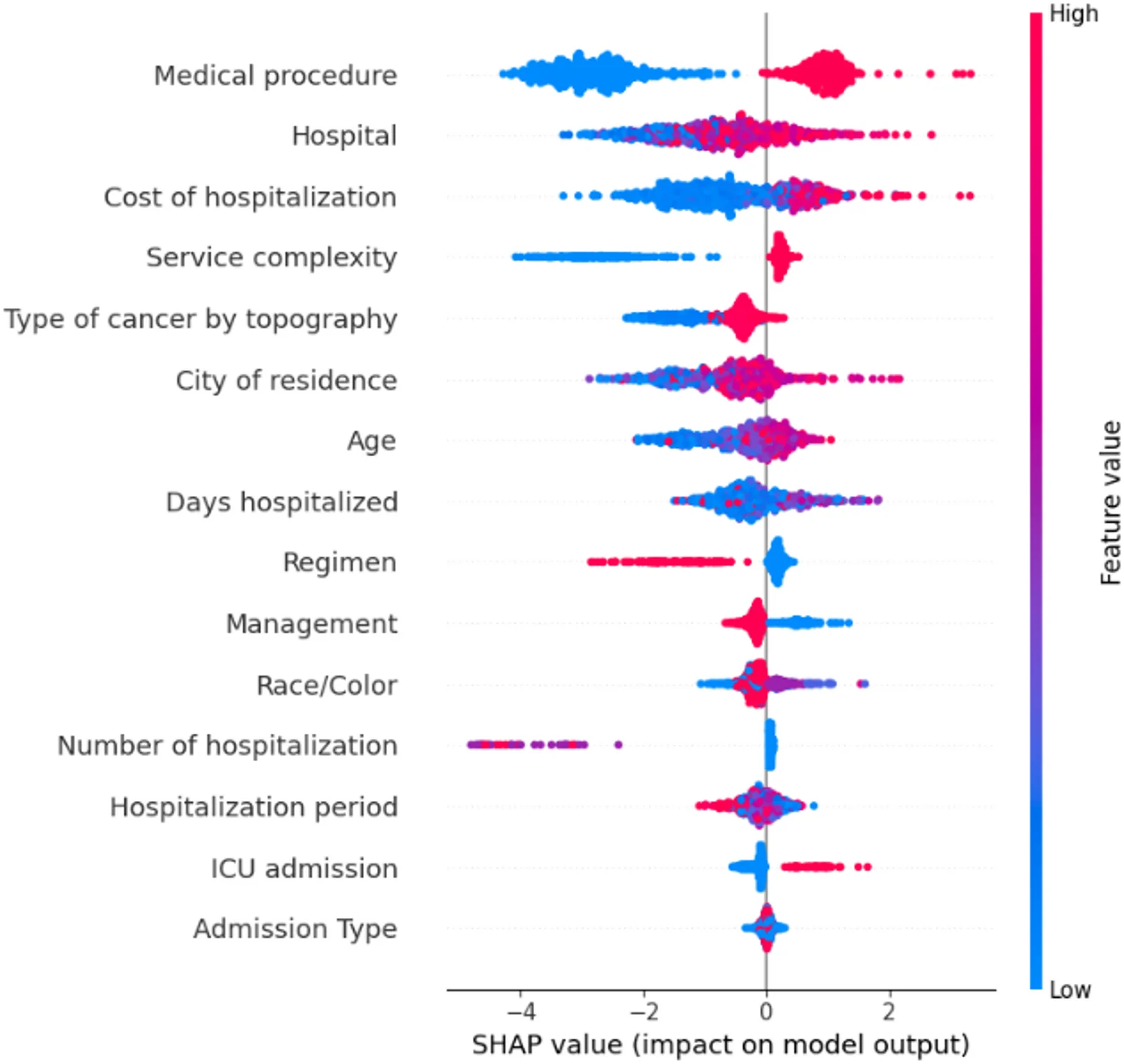
Contribution of predictors in hospital mortality in cervical cancer: SHAP plot using XGBoost algorithm

**Fig. 3:**
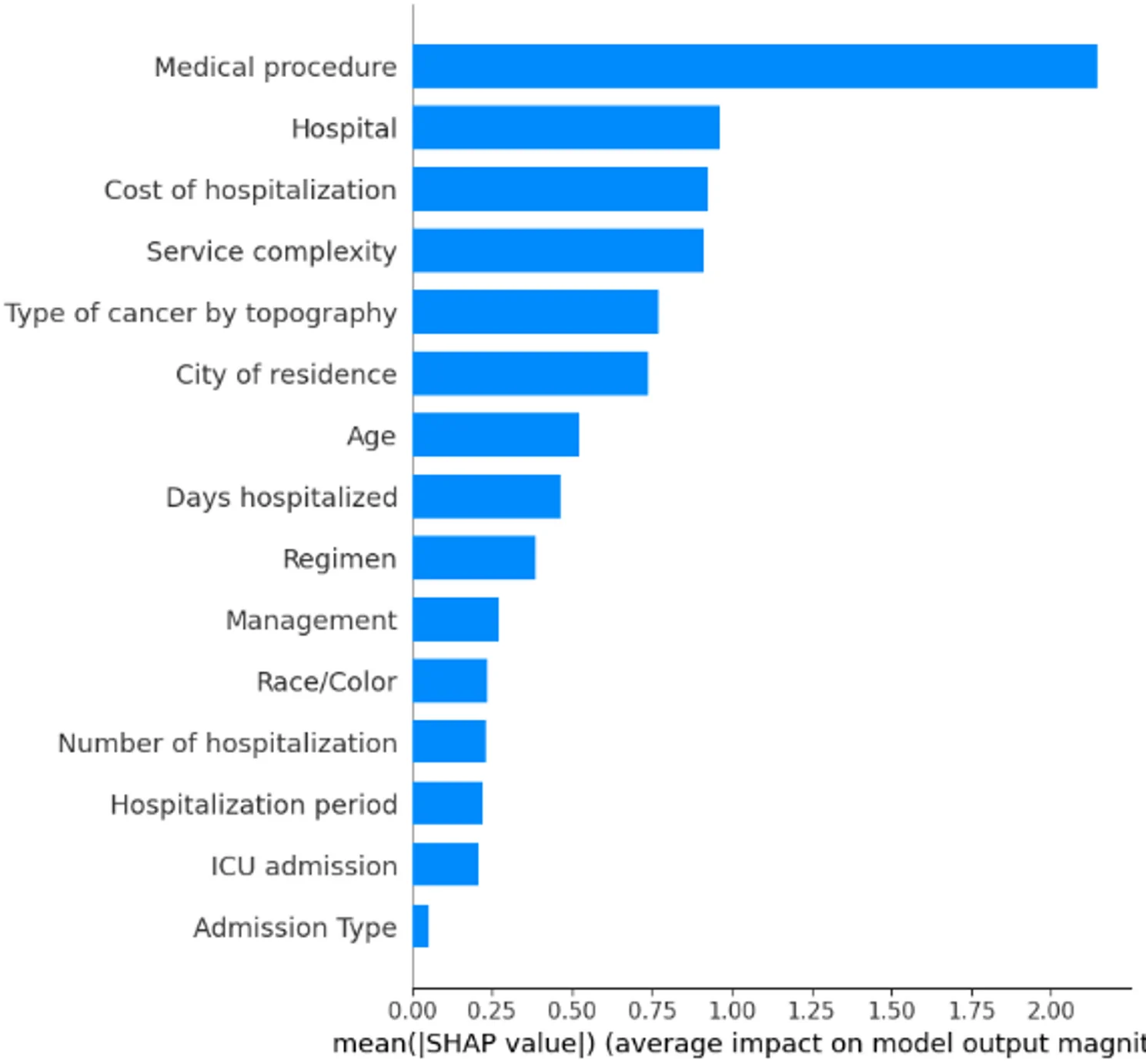
Variable importance for hospital mortality in cervical cancer: XGBoost algorithm on test set

SHAP values were used to examine the contribution of individual predictors to the XGBoost model’s outputs on the test set. The SHAP summary plot **(**Fig. 2**)** identified medical procedure type, hospitalisation cost, and service complexity as the most influential predictors of in-hospital mortality, with strong separation in SHAP values indicating their central role in distinguishing survivors from non-survivors. Admission type and hospitalisation period had moderate contributions, whereas age and race/skin colour had only minimal impact on model outputs. Higher hospitalisation costs were associated with higher predicted probabilities of mortality, whereas surgical procedures were associated with lower predicted mortality compared with clinical admissions. In addition to the SHAP-based analysis, the relative importance of predictors in the XGBoost model was examined using the gain-based variable importance measure (Fig. 3). An individual-level explanation is provided by the SHAP force plot in Fig. S4, which illustrates how feature contributions combined to produce a low-mortality prediction (f(x) = - 12.55) for a single patient, substantially below the model’s base value (0.00627).

Model calibration was further examined using reliability diagrams (Fig. S5). The tree-based models were well calibrated, closely following the diagonal of perfect calibration across the range of predicted probabilities in which most patients lay, whereas Logistic Regression deviated below the diagonal and over-estimated mortality risk, consistent with its higher Brier score. After post-hoc isotonic recalibration, all models were well calibrated and their Brier scores converged to approximately 0.07-0.08. Decision curve analysis (Supplementary Fig. S6) showed that, across the clinically relevant range of threshold probabilities (approximately 0.02-0.40), all ML models provided a higher net benefit than the default ‘treat-all’ and ‘treat-none’ strategies, with the tree-based models offering the greatest net benefit.

## Discussion

The results of this study underscore the significant potential of ML models in predicting hospital mortality among CC patients, particularly in resource-constrained regions. The superior performance of the XGBoost model (88% accuracy, 95% specificity, and an AUC-ROC of 0.89) highlights its ability to identify complex data patterns, corroborating prior research on the efficacy of ML algorithms in oncology and clinical outcome prediction [13, 15, 18, 20]. These findings are particularly relevant in low- and middle-income settings, where the precision of predictive models can enhance resource allocation and patient management [17]. Importantly, the value of this approach lies not in identifying novel clinical risk factors, but in quantifying and integrating multiple clinical and system-level variables within a heterogeneous real-world hospitalised population.

CC remains a significant contributor to cancer mortality among women in developing countries. Socioeconomic disparities and limited access to specialized care negatively affect clinical outcomes, as corroborated by this study, which identified advanced age, prolonged hospitalisation, and ICU admission as key predictors of mortality. These factors have been similarly associated with poor outcomes in other studies focusing on advanced cancer patients [6, 9]. Although these predictors may appear clinically intuitive when considered individually, their combined and nonlinear contributions to mortality risk are difficult to quantify using traditional analytical approaches. Addressing these predictors through targeted interventions, particularly in ICU settings, could help reduce mortality rates in vulnerable populations.

Compared to traditional statistical methods such as logistic regression, tree-based ML models like XGBoost, Random Forest, and LightGBM are better equipped to capture complex interactions and nonlinear patterns within data [35, 36]. This capability is particularly beneficial in clinical settings where multiple factors interact to influence patient outcomes. In this study, ML models were used to accommodate the inherent heterogeneity of hospitalised cervical cancer patients, including those admitted for curative procedures and those hospitalised for complications of advanced disease, reflecting real-world care pathways rather than artificially homogeneous cohorts. However, the effective translation of these insights into practice requires investment in infrastructure and specialized training for healthcare professionals, a challenge in low-income regions [7].

A key strength of this study is its use of SHAP to interpret model outputs. SHAP provides a transparent framework for identifying and ranking predictors, bridging the gap between algorithmic complexity and clinical usability [31, 32, 37]. In this study, predictors such as medical procedure type, hospitalisation cost, and service complexity emerged as critical factors, while traditional variables like age and race played minor roles. Rather than serving as tools for early bedside decision-making, these predictors function as proxies for disease severity, care complexity, and resource utilisation within the hospital system. By capturing these nuanced patterns, SHAP-based ML models provide actionable insights to guide retrospective risk stratification, health system evaluation, and benchmarking of hospital performance, particularly in settings with constrained healthcare infrastructure.

Our findings align with recent research on ML applications in oncology [12, 17, 38]. For instance, Saini et al. (2021), demonstrated that ML algorithms are particularly useful for predicting adverse outcomes in oncology, especially when large volumes of clinical data are available [13]. Other studies, like Topol’s, emphasise that artificial intelligence has the potential to transform healthcare delivery, but its implementation requires robust infrastructure and the trust of healthcare professionals [16]. This study develops and evaluates ML models for hospital-level in-hospital mortality risk in cervical cancer using national administrative data. Our models (AUC-ROC 0.87-0.89) perform within the typical range for ML-based mortality models (0.80-0.90) and add disease-specific interpretability via SHAP. Unlike models based on admission physiological data or ICU cohorts, ours use routine administrative variables from the Brazilian SUS to support hospital benchmarking, resource allocation, and health system evaluation in similar low- and middle-income settings, not bedside decisions.

Beyond discrimination and calibration, the decision curve analysis indicates that model-guided triage could yield a positive net benefit relative to broad default strategies across a wide range of threshold probabilities, which is particularly relevant for allocating scarce resources in settings such as Mato Grosso. A formal cost-effectiveness evaluation was beyond the scope of this study and represents an important direction for future work.

Despite its strengths, this study has limitations. First, the use of data from the SIH, an administrative hospitalisation database, restricts the availability of detailed clinical information such as cancer staging, comorbidities, and complete treatment histories, which may reduce the clinical granularity of the predictive models [21]. In addition, variables are recorded at the level of the complete hospitalisation episode rather than at admission, meaning that some predictors, particularly hospitalisation cost, reflect events and resource use accumulated throughout the hospital stay, limiting the applicability of the model for early bedside or admission-time decision-making. Second, hospitalisations rather than individual patients were used as the unit of analysis. As a result, patients with multiple admissions may have contributed more than one observation, with each hospitalisation treated as an independent event. This approach, while consistent with prior studies using SIH data, precluded the assessment of disease duration and longitudinal clinical trajectories, which are relevant in chronic conditions such as cervical cancer. Additionally, missing data were handled using mean imputation, a pragmatic but relatively simple approach that may not fully capture the underlying variability of the variables. Although this strategy supports transparency and reproducibility when working with large administrative datasets, more advanced imputation methods could be explored in future studies.

The class imbalance in the dataset, with only 9.9% of patients classified as non-survivors, also posed challenges. While XGBoost achieved high specificity and AUC-ROC, the MCC was relatively low. This metric is sensitive to class imbalances and may underestimate the model’s performance in predicting minority outcomes. This finding underscores the importance of evaluating multiple metrics to provide a comprehensive assessment of model performance.

This study shows interpretable ML models can support hospital risk stratification and health system assessment of cervical cancer care in resource-limited settings. Using SHAP to transparently attribute predictions to clinical and system variables, these models aid in retrospective quality monitoring, hospital benchmarking, and resource allocation, rather than bedside clinical use. When integrated into management workflows, they can identify hospitals or patient groups with high in-hospital mortality and guide targeted interventions.

To further advance this field, future studies should prioritise external validation using diverse datasets to account for variability in healthcare practices and patient demographics. Incorporating additional clinical variables, such as cancer staging and treatment histories, could enhance the predictive accuracy and generalizability of the models. Additionally, temporal validation is recommended to assess model performance over time and adapt to changes in healthcare systems. These steps are essential for ensuring the robustness and scalability of ML-based solutions in clinical oncology.

## Conclusion

This study shows that ML models built on routinely collected administrative hospitalisation data can support hospital-level risk stratification of in-hospital mortality in cervical cancer, especially in settings with limited healthcare infrastructure. Rather than functioning as bedside prediction tools, these models are positioned as instruments for health system evaluation, hospital benchmarking, and resource allocation in low- and middle-income regions. Future work should focus on external and temporal validation across different states and databases, and on incorporating additional clinical variables, such as cancer staging and treatment histories, to refine the models and broaden their applicability for management and policy use.

## Supplementary Information

Supplementary Material 1.

## Data Availability

The datasets analyzed in this study are publicly available through the Mato Grosso State Health Information System (SIH).
