# Supplementary material for: Machine learning for risk stratification of in-hospital mortality in patients with cervical cancer: an analysis of hospital admissions in Mato Grosso, Brazil
Source: BMC Cancer. 2026 Aug 18;26:1410. doi: 10.1186/s12885-026-16771-z (PMC13761170; doi:10.1186/s12885-026-16771-z)
Supplement: Supplementary file 1 [file 12885_2026_16771_MOESM1_ESM.docx]

**Supplementary Material**

**Fig S1. Correlation matrix of predictors used in the models**

**Fig S2. Confusion matrix of hospital mortality prediction in cervical cancer for test data across algorithms.**

**Fig S3.** **Methodology workflow for predicting hospital hortality in CC Patients**

**Table S1. Model performance across different probability thresholds**

| **Model** | **Threshold** | AUC-ROC (95% CI) | **Sensitivity** | **Specificity** | **Precision** | **F1** |
| --- | --- | --- | --- | --- | --- | --- |
| **Logistic Regression** | 0.2 | 0.88 (0.85–0.91) | **0.962** | 0.653 | 0.231 | 0.373 |
|  | 0.3 | 0.88 (0.85–0.91) | 0.885 | 0.735 | 0.266 | 0.410 |
|  | 0.4 | 0.88 (0.85–0.91) | 0.833 | 0.767 | 0.280 | 0.419 |
|  | 0.5 | 0.88 (0.85–0.91) | 0.821 | 0.781 | 0.290 | **0.428** |
| **Random Forest** | 0.2 | 0.88 (0.85–0.90) | 0.859 | 0.770 | 0.289 | 0.432 |
|  | 0.3 | 0.88 (0.85–0.90) | 0.692 | 0.847 | 0.329 | **0.446** |
|  | 0.4 | 0.88 (0.85–0.90) | 0.513 | 0.903 | 0.364 | 0.426 |
|  | 0.5 | 0.88 (0.85–0.90) | 0.321 | **0.936** | 0.352 | 0.336 |
| **CatBoost** | 0.2 | 0.88 (0.85–0.90) | 0.551 | 0.897 | 0.368 | **0.441** |
|  | 0.3 | 0.88 (0.85–0.90) | 0.449 | 0.919 | 0.376 | 0.409 |
|  | 0.4 | 0.88 (0.85–0.90) | 0.385 | 0.933 | **0.385** | 0.385 |
|  | 0.5 | 0.88 (0.85–0.90) | 0.333 | **0.942** | 0.382 | 0.356 |
| **LightGBM** | 0.2 | 0.87 (0.84–0.90) | 0.500 | 0.908 | 0.371 | **0.426** |
|  | 0.3 | 0.87 (0.84–0.90) | 0.397 | 0.930 | **0.383** | 0.390 |
|  | 0.4 | 0.87 (0.84–0.90) | 0.333 | 0.936 | 0.361 | 0.347 |
|  | 0.5 | 0.87 (0.84–0.90) | 0.218 | **0.940** | 0.283 | 0.247 |
| **XGBoost** | 0.2 | 0.89 (0.86–0.91) | 0.603 | 0.882 | 0.356 | **0.448** |
|  | 0.3 | 0.89 (0.86–0.91) | 0.513 | 0.911 | **0.385** | 0.440 |
|  | 0.4 | 0.89 (0.86–0.91) | 0.385 | 0.930 | 0.375 | 0.380 |
|  | 0.5 | 0.89 (0.86–0.91) | 0.308 | **0.947** | 0.387 | 0.343 |

 **Fig S4. SHAP force plot showing individual-level contributions to hospital mortality prediction in a cervical cancer patient**

This SHAP force plot illustrates the contribution of each variable to the prediction made by the machine learning model for a single cervical cancer patient. The predicted model output *f(x)=−12.55*, which is far below the base value (0.00627), suggests a very low predicted probability of hospital mortality. Variables such as absence of medical procedures, low cost of hospitalization, low service complexity, and hospial and geographic characteristics reduced the estimated risk (blue). This visual explanation highlights how specific factors influence the model’s individual prediction.

**Fig S5. Calibration curves of hospital mortality prediction across algorithms, before and after isotonic recalibration.**

Predicted probabilities are plotted against the observed fraction of in-hospital deaths in the test set; the dashed line denotes perfect calibration. Left panel (before recalibration): the tree-based models (XGBoost, Random Forest, CatBoost and LightGBM) follow the diagonal reasonably well across the range of predicted probabilities in which most patients lie, whereas Logistic Regression falls below the diagonal and over-estimates mortality risk, consistent with the Brier scores in Table 2. Right panel (after post-hoc isotonic recalibration, fitted on a held-out calibration subset of the training data): all five models are well calibrated, closely following the diagonal, with Brier scores converging to approximately 0.07–0.08.

**Fig S6. Decision curve analysis of hospital mortality prediction across algorithms.**

Net benefit of each model is plotted against the threshold probability and compared with the default ‘treat-all’ and ‘treat-none’ strategies. Across the clinically relevant range of threshold probabilities (approximately 0.02–0.40), all machine-learning models yield a higher net benefit than both default strategies, with the tree-based models providing the greatest net benefit. Decision curve analysis supports the potential clinical utility of the models for model-guided risk stratification and resource allocation, without requiring a full health-economic model.
